# The Story of the Insane from Year to Year

**Published:** 1900-05-12

**Authors:** 


### The Institutional Workshop.

### THE STORY OF THE INSANE FROM YEAR TO YEAR.

Twelfth Series.

### THE NORFOLK COUNTY ASYLUM AT NORWICH.

In this asylum the numbers on January 1st, 1898, were 831, and on December 31st 854. There were 157 first admissions, 37 readmissions, 56 recoveries, and 99 deaths. The recoveries stand at 30·72 per cent. on the admissions. This percentage is much below the average in county asylums generally, and also below the Norfolk Asylum average. The deaths stand at 11·91 per cent. calculated on the average number resident. Of these deaths 41 were caused by cerebral and spinal diseases, 14 by consumption, 6 by bronchitis, 6 by pneumonia, and 1 by colitis. The latter seems to have been the only case of zymotic disease, and it was not contracted in the asylum. Of the year's admissions 41 owed their insanity to moral causes, 7 to intemperance in drink, 90 to hereditary influence, and in 132 cases the cause is put down as unknown, but this figure must be a mistake, as there were only 194 admissions. It speaks well for the management of the asylum that there has not been a case of suicide in it for twelve years, although at least 25 per cent. of the admissions during that period must have had suicidal tendencies. Dr. Thomson and his medical staff began to train the nurses in 1896, and about one-third of the nursing staff now hold the nursing certificate of the Medico Psychological Association. We should be glad to hear in the next annual report that Dr. Thomson has inaugurated a three years' course of instruction for his staff. The committee record "their satisfaction with the efficient manner in which Dr. Thomson has managed the establishment." The average weekly cost per head is 9s. 0½d.

---

### THE BARNWOOD HOUSE HOSPITAL FOR THE INSANE AT GLOUCESTER.

This hospital contained on January 1st, 1898, 155 patients and 161 on December 31st. There were 30 first admissions, 4 readmissions, 13 recoveries, and 6 deaths. Calculated on the admissions of the year the recoveries stand at 38·2 per cent., and, calculated on the average number resident, the deaths stand at 4·4 per cent. The recoveries are a little over the general average for the whole of England for 1898, and the deaths are much below the average; but in making any comparison it is essential to remember that the class of patients admitted to Barnwood differs materially from that admitted to our county asylums. Of the deaths during the year 2 were caused by general paralysis, 1 from melancholic exhaustion, 1 from convulsions, and 2 from senile decay. Of the admissions 11 owed their insanity to moral causes, 3 to drink, and 14 to hereditary influence. Dr. Soutar says that in not more than 8 of the year's admissions was there any prospect of recovery, and he regrets that he is called upon to receive so large a proportion of the chronic insane. If his remark refers to those admitted at greatly reduced rates, or at no payments, we do not agree, because it is often possible for the friends of the insane to make an effort to find money sufficient to pay for six months' residence in a private asylum for an acute case, whereas it is with the chronic cases that the pinch so often comes, and surely it must, or ought, to be for these cases that the "chronic" part of the hospital exists. A comfortable home for these cases is precisely what they require, and if the arrangements have gone beyond what is necessary for this it is scarcely the fault of the patient. There has been no restraint used during the year, and only two patients have been secluded for short periods. We are glad to see that Dr. Soutar occasionally uses seclusion, as it is undoubtedly a useful means of supplementing other treatment. The general management of the asylum is believed to be conducted on a most liberal basis; the entertainments are very numerous; parties of patients often go to Gloucester and Cheltenham to concerts and lectures, and for those who need a change of air a residence is kept in the Forest of Dean. The female division contains only 98 patients, and for these are provided a matron, a head nurse, eight lady companions, 23 nurses, four pantry maids, and three housemaids. We learn that one patient was admitted free of charge, and ten below the cost of their maintenance, namely, at from £1 to £1 10s. a week; and we further learn, this time with regret, that 12 applications were refused admittance at reduced rates. These patients were suffering from chronic insanity. We failed to see any table showing the number of patients kept free of charge and the number kept at reduced rates. The visitors say that Dr. Soutar "possesses the entire confidence of the committee," and that he "has displayed the same assiduity and administrative ability as in past years."

